# A Tensile Deformation Model for *In-situ* Dendrite/Metallic Glass Matrix Composites

**DOI:** 10.1038/srep02816

**Published:** 2013-10-02

**Authors:** J. W. Qiao, T. Zhang, F. Q. Yang, P. K. Liaw, S. Pauly, B. S. Xu

**Affiliations:** 1 Laboratory of Applied Physics and Mechanics of Advanced Materials, College of Materials Science and Engineering, Taiyuan University of Technology, Taiyuan 030024, China; 2 Research Center of Advanced Materials Science and Technology, Taiyuan University of Technology, Taiyuan 030024, China; 3 Department of Chemical and Materials Engineering, University of Kentucky, Lexington, KY 40506, USA; 4 Department of Materials Science and Engineering, The University of Tennessee, Knoxville, TN 37996-2200, USA; 5 IFW Dresden, Institut für Komplexe Materialien, Helmholtzstraβe 20, D-01069 Dresden, Germany

## Abstract

*In-situ* dendrite/metallic glass matrix composites (MGMCs) with a composition of Ti_46_Zr_20_V_12_Cu_5_Be_17_ exhibit ultimate tensile strength of 1510 MPa and fracture strain of about 7.6%. A tensile deformation model is established, based on the five-stage classification: (1) elastic-elastic, (2) elastic-plastic, (3) plastic-plastic (yield platform), (4) plastic-plastic (work hardening), and (5) plastic-plastic (softening) stages, analogous to the tensile behavior of common carbon steels. The constitutive relations strongly elucidate the tensile deformation mechanism. In parallel, the simulation results by a finite-element method (FEM) are in good agreement with the experimental findings and theoretical calculations. The present study gives a mathematical model to clarify the work-hardening behavior of dendrites and softening of the amorphous matrix. Furthermore, the model can be employed to simulate the tensile behavior of *in-situ* dendrite/MGMCs.

Due to their unique properties, including exceptionally high strength, elastic limit, and hardness, excellent corrosion resistance, reduced sliding friction, improved wear resistance *etc*.^1^,^2^,^3^, bulk metallic glasses (BMGs) are regarded as potential candidate materials in engineering fields. However, their structural applications are severely stymied by the prevalence of low ductility and brittle fracture upon loading at room temperature. Lack of pronounced macroscopic plasticity of BMGs are correlated with highly localized shear banding, and a great amount of plastic strain is accumulated in narrow shear bands, exhibiting strain softening by adiabatic shearing^4^. Even though the local plastic strain in shear bands is very high, the overall room-temperature plastic deformation is disappointingly low.

To circumvent the poor damage tolerance of BMGs, several strategies have been adopted to improve the room-temperature ductility in BMGs, including microstructure modification by adding dispersive *ex-* and *in-situ* reinforcements in the amorphous matrix to form dual-phase composites^1^,^5^,^6^,^7^,^8^, surface modifications, such as shot peening^9^, and molding optimal microstructure architectures^10^, and composition designs with ‘soft’ and ‘hard’ regions^11^. These approaches aim to create a more homogeneous distribution of shear bands and make shear band multiplication^12^, so that the formation of detrimental widely-spaced shear bands or single shear bands leading to early failure is effectively hindered. Since the amorphous structure is unchanged, the ductility of monolithic BMGs through special treatments increases distinguishingly less than the introduction of secondary ductile phases.

The dual-phase metallic glass matrix composites (MGMCs) were firstly fabricated through an *ex-situ* processing, by which solid crystalline phases were added to the molten matrix^13^. Later, several groups^1^,^6^,^14^,^15^,^16^,^17^,^18^ developed *in-situ* MGMCs, in which ductile crystalline phases nucleated and grew to form a solid solution during the process of cooling from the melt. Thereinto, ductile dendrite/metallic glass matrix composites, with a homogeneous distribution, high glass-forming ability of matrices, and improved toughness, have been widely developed to solve the conflict between strength and toughness^6^,^17^,^18^. In these *in-situ* MGMCs, the amorphous matrix provides extreme strength, while the dendrites can apparently suppress catastrophic failure due to shear localization, and lead to legitimate plastic flows. However, it should be noted that most of the developed *in-situ* MGMCs exhibit softening upon tension rather than work hardening upon compression^6^, giving an implication that the tensile mechanism may be very different from the corresponding compressive one. The challenge for structural applications is how to obtain the tensile ductility and work-hardening capacity. Only if the materials can be homogeneously plastically deformed, the localized deformation and softening leading to the early failure can be avoided. Up to now, the detailed tensile mechanism for *in-situ* MGMCs remains poorly understood. In this study, we explored the tensile mechanisms, based on the theoretical calculations and finite-element method (FEM) analysis.

## Results

**Figure 1:**
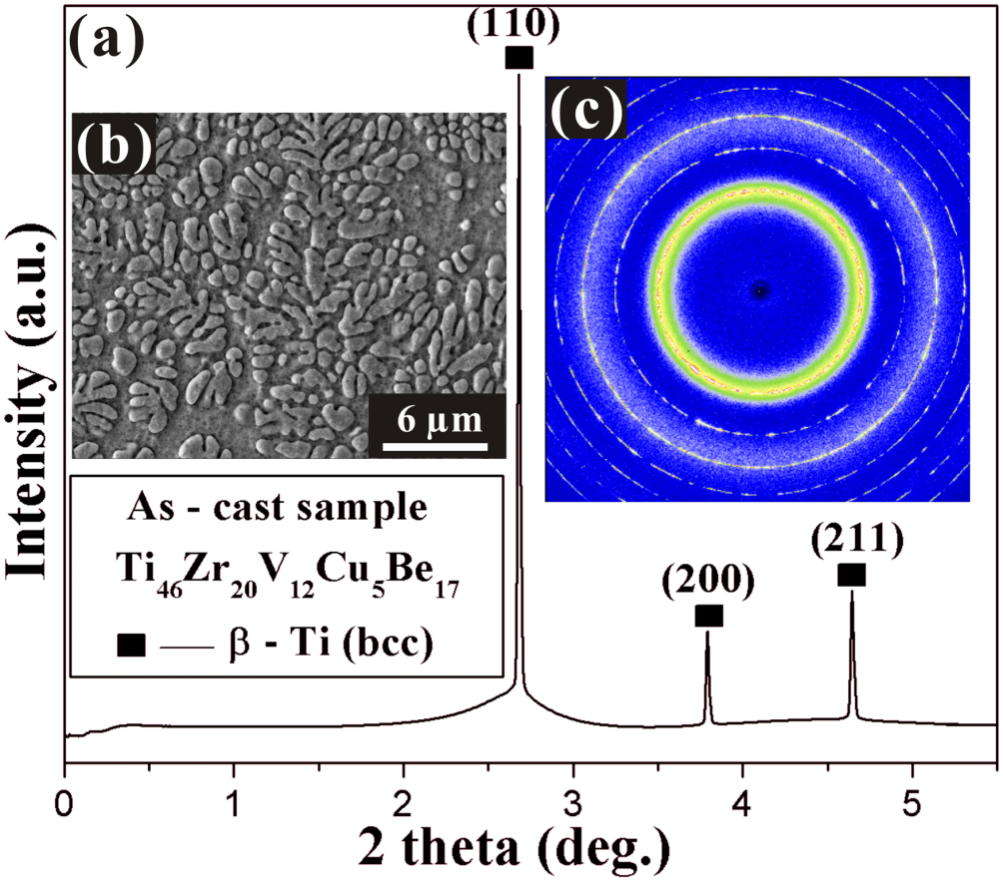
The high-energy synchrotron X-ray diffraction pattern of the composite in (a); the micrograph of the as-cast composite shown in (b); and diffraction rings, corresponding to the X-ray diffraction pattern, shown in (c).

Figure 1(a) shows the high-energy synchrotron X-ray diffraction pattern of the composite with a nominal composition of Ti_46_ Zr_20_V_12_Cu_5_Be_17_. Sharp diffraction peaks of crystalline phases, which is a body-centered cubic (bcc) β solid solution, as well as broad and diffused patterns of the amorphous phase are found within the composites, indicating the presence of crystalline phases in the amorphous matrix. The micrograph of the as-cast composite is shown in Figure 1(b). It can be seen that the dendrites are well developed in the composite, and uniformly distributed within a featureless glass matrix. The volume fraction of the dendrites is approximately 57%, and the average size of dendrites is about 2 μm. The energy-dispersive-spectrometer (EDS) analysis gives the average atomic composition (at.%) of the amorphous matrix, Ti_44.04_Zr_7.7_V_16.2_ Cu_1.35_Be_23.72_, under the assumption that all of the element of Be is partitioned into the glass matrix, the average atomic composition (at.%) of the dendrites can be estimated at Ti_43.31_Zr_43.44_V_1.16_ Cu_12.09_. The DSC trace of the present MGMC shows a glass-transition behavior and supercooled liquid regions, suggesting that the alloy containing amorphous phases. Fully continuous diffraction rings shown in Figure 1(c), corresponding to the X-ray diffraction pattern, is ascribed to the homogeneously-distributed dendrites.

**Figure 2:**
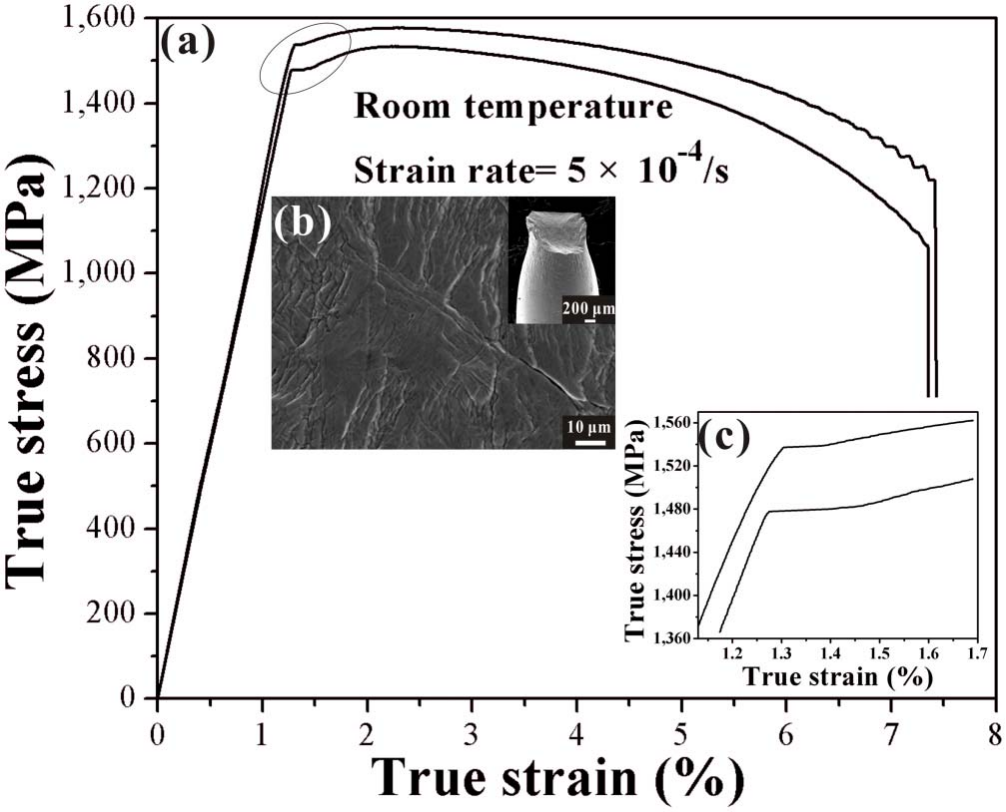
The true stress-strain curves of the present composites upon tension in (a); the fractography of the present composite after tension in (b) and macroscopic necking in the inset of (b); and the yielding platform, marked by the oval in (a), shown in (c).

Figure 2(a) shows the true stress-strain curves of the present composites upon tension. It can be seen that the results of duplicate tests are very consistent. The curves are analogous to the stress-strain curves of the traditional carbon steels (yielding platform, work hardening, and softening), and the similar phenomenon has been found for an *in-situ* composite with the volume fraction of dendrite of 43%^19^. Conservatively, the stress-strain curve with a lower strength in Figure 2(a) is used to estimate the mechanical properties of the present composites. The yielding strength and yielding strain are ~1460 MPa and ~1.23%, respectively. The tensile strength and fracture strain are ~1510 MPa and ~7.6%, respectively. Figure 2(b) shows the fractography of the present composite after tension. Profuse shear bands are distributed on the lateral surface of deformed samples, and macroscopic necking can be observed in the inset of Figure 2(b), in agreement with the large tensile ductility. The yielding platform of the present composite, marked by the oval in Figure 2(a), is presented in Figure 2(c).

## Discussion

**Figure 3:**
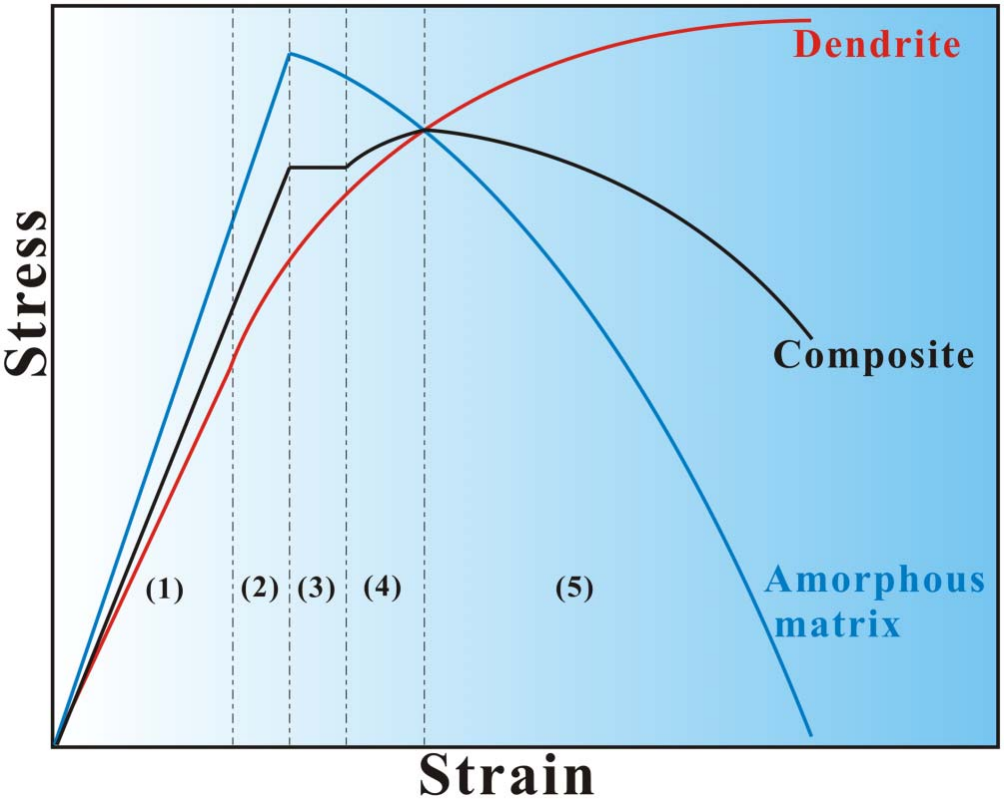
Illustration of (1) elastic-elastic, (2) elastic-plastic, (3) plastic-plastic (yield platform), (4) plastic-plastic (work hardening), and (5) plastic-plastic (softening) stages of the dendrites, amorphous matrix, and composite.

Usually, under compression, the work hardening of *in-situ* dendrite/MGMCs prevails until the final fracture once the yielding happens^1^,^16^,^17^. In contrast, little work hardening accompanied by remarkable softening is gained for *in-situ* dendrite composites upon tension^6^,^18^,^19^. According to Figure 2(a), the tension behavior of the present *in-situ* dendrite/MGMCs is classified into five stages: (1) elastic-elastic, (2) elastic-plastic, (3) plastic-plastic (yield platform), (4) plastic-plastic (work hardening), and (5) plastic-plastic (softening) stages, as schematically illustrated in Figure 3. In Figure 3, it is suggested that the amorphous matrix would exhibit large ductility and macroscopic necking, since mature shear banding would be frequently interrupted due to the existence of many large plastic zones ahead of cracks.

In the first stage, both the dendrites and amorphous matrix are elastic, and the composite is also under elastic loading. The stress-strain relations of the amorphous matrix and ductile dendrites are expressed as follows^19^,^20^: 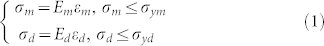
where *E_m_* and *E_d_* are Young's moduli of the amorphous matrix and dendrites, respectively, 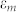 and 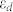 are the elastic strains of the amorphous matrix and dendrites, respectively, and 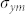 and 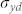 are the tensile yield stresses of the amorphous matrix and dendrites, respectively. It should be noted that 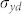 is determined as the stress for the occurrence of shear banding on the macroscopic scale. *E_m_* and *E_d_* obtained from the nanoindentation measurement are 130.4 and 106.3 GPa, respectively. 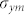 and 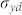 are suggested to be 1680^21^, and 1336 MPa^22^, respectively.

The Young's modulus of the composite, *E_c_*, can be estimated according to Hashin and Shtrikman^23^: 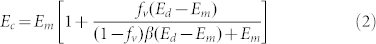
where *f_v_* is the volume fraction of dendrites with a value of 0.57, *β* is the material constant calculated by 
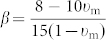
, and 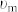 is the Poisson's ratio of the amorphous matrix with a value of about 0.352 21,24. From Eq. (2), the *E*_c_ value is found to be 116.2 GPa, which is very close to the experimental value of 113.2 GPa obtained from the stress-strain curves in Figure 2(a).

In addition, a simple rule of mixture is employed as a first-order approximation to evaluate the axial stress of the composite, 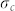, highly dependent on the stress of dendrites, 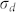, and the stress of amorphous matrix, 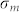: 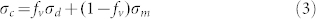
It is noted that the upper and low boundaries during evaluation of the strength of *in-situ* composites are not considered in the present study, and the simple approximation is adopted. For elastic deformation, it is suggested that 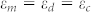 (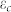 is the strain of the composite), since elastic deformation is very small, and the misfit of both phases will not lead to large strain dissimilarity. Using Eq. (1), Eq. (3) can be simplified as: 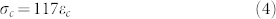
It should be noted that the unit of the stress in the present analysis is MPa. With further straining, the weaker phase starts to deform plastically first. The bcc Ti alloys usually have a lower yielding stress than the Ti-based BMGs^22^,^25^,^26^,^27^. Consequently, the dendrites yield first. The second stage commences. The amorphous matrix deforms elastically, while the dendrites deform plastically^19^. The plastic strain in the dendrites can be calculated according to a Taylor dislocation model^28^, and the tensile stress-strain relation in the dendrites is given as: 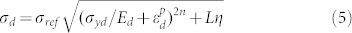
where 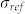 is the reference stress of ductile dendrites upon uniaxial tension, and 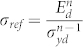. Here 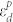 is the plastic strain of the dendrites, *n* is the hardening coefficient of the dendrites with the value of about 0.07 29,30, and 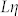 represents the contribution to the strain hardening from geometrically necessary dislocations [*L* is the intrinsic material length of the ductile phase, and 
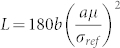
 28]. 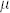 and *b* are the shear modulus and Burgers vector of the dendrites, and *a* is an empirical material constant in the Taylor dislocation model ranging from 0.1 to 0.5 31. In this work, *a* is selected to be 0.3 in a mediate value, and 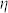 is the effective plastic-strain gradient, which can be replaced by an average plastic strain gradient, 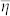. Here, 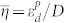, where *D* is the average diameter of the dendrites.

The shear modulus of the dendrites, 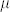, is calculated by 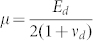, 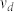 is the Poisson's ratio of the dendrites with a value of about 0.33 22,25,32. Assume that the Burgers vector of the dendrites, *b*, is about 1 nm. Eq. (5) can be rewritten as: 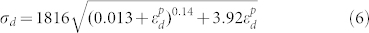
The contribution of the working hardening of the dendrites to the strength of the composite can be expressed by 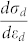. The differential equation from Eq. (6) is as follow: 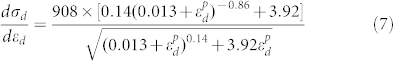
As the dendrites yield, the relationship between the tensile strain of composite, 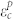, and that of dendrites is given by^33^: 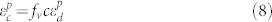
where *c* is the average stress concentration factor of the dendrites with an approximate value of 1 20. Eq. (8) can be rewritten as 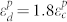.

Assuming 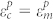, from Eqs. (1), (3), (6), and (8)Eqs. (1), (3), (6), and (8)Eqs. (1), (3), (6), and (8)Eqs. (1), (3), (6), and (8), the constitutive relation in the second stage can be expressed as: 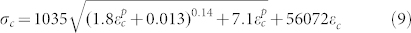
Once the tensile stress approaches the yield strength of the glass matrix, both phases deform plastically, i.e., the dendrites exhibit work hardening, and the shear bands start to initial and propagate in the amorphous matrix, accompanied by the increase of the shear offset and accommodation of localized plastic deformation^19^,^34^. Assme that shear bands initiate and propagate under the resolved 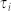 along the 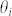 direction, and the contribution of the plastic shear strain, 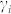, to global plastic tensile strain is 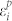. The accumulated plastic strain during multiplication of shear bands can be calculated. The relationship between the tensile stress and shear stress, and that between tensile strain and shear strain can be expressed as follows: 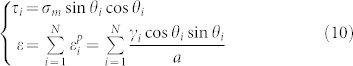
where *a* is the ratio of the length to width in the gauge portion, and *N* is the number of shear bands that can propagate under the tensile stress, 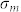, applied to the composite.

It is well known that if the monolithic BMG starts to yield, it will quickly enter softening deformation stage. As both phases deform plastically, an approximate yielding platform is present, as shown in Figure 2(c), analogous to previous results^19^. Neither work hardening nor softening is dominating. In this third stage, the two phases are under plastic deformation. The contribution from work hardening of dendrites and softening of the amorphous matrix be equal, i.e. 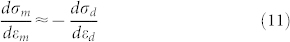
Here, 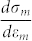 can be considered as the contribution from the softening behavior of the amorphous matrix to the strength of the composite.

Combining Eqs. (7) and (11)Eqs. (7) and (11), and assuming 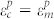, one obtains: 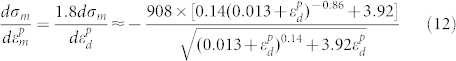
The integration of the Eq. (12) yields the following relation: 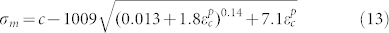
where *c* is a constant, and is obtained from the stress-strain curve in Figure 2(a) with a value of 2463 MPa.

From Eq. (10), it is very difficult to obtain a quantitative calculation. By fitting the curves of the fourth stage (work hardening) from the stress-strain curves in Figure 2(a), the constitutive relation in the fourth stage can described as: 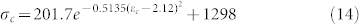
Combining Eqs. (3), (6), (8), and (14)Eqs. (3), (6), (8), and (14)Eqs. (3), (6), (8), and (14)Eqs. (3), (6), (8), and (14), the relationships between the tensile stress and tensile strain of the amorphous matrix is given as: 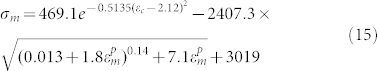
From Eqs. (14) and (15)Eqs. (14) and (15), the composite in the fourth stage shows little work hardening with 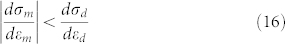
By fitting the curve of the fifth stage, the constitutive relation of the composite can be expressed as: 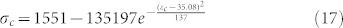
From Eqs. (3), (6), (8), and (17)Eqs. (3), (6), (8), and (17)Eqs. (3), (6), (8), and (17)Eqs. (3), (6), (8), and (17), the deformation of the amorphous matrix in the fifth stage can be described as: 
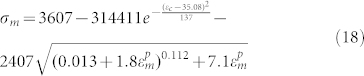
In the fifth stage, from Eqs. (17) and (18)Eqs. (17) and (18), the composite starts to soften. In this stage, 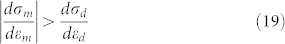
Regardless of the fourth or fifth stage, it has been demonstrated that the interaction between the crystalline second phase and the localized shear bands is dominated by the cooperative activation of lattice dislocations on the glass–crystal interfaces and discrete shearing events in the neighboring glass matrix^35^. Once the fifth stage is available, although the stress within the localized necking part continues to increase, the resistance to hardening is evidently decreased. As a result, the softening of the composite happens, dominated by shear banding^36^. Close to the final fracture, the serration on the stress-strain curve gives an evidence of domination of the shear banding on the amorphous matrix, covering the work hardening of dendrites.

Based on the theoretical model, the constitutive relations are established in each deformation stage, which gives quantitative characterization. Parallel to the characterization of deformation model, FEM analysis is used not only to testify the classification of the tensile deformation stages for *in-situ* dendrite/MGMCs, but also to elucidate the stress evolution upon the tensile deformation of dual-phase MGMCs.

**Figure 4:**
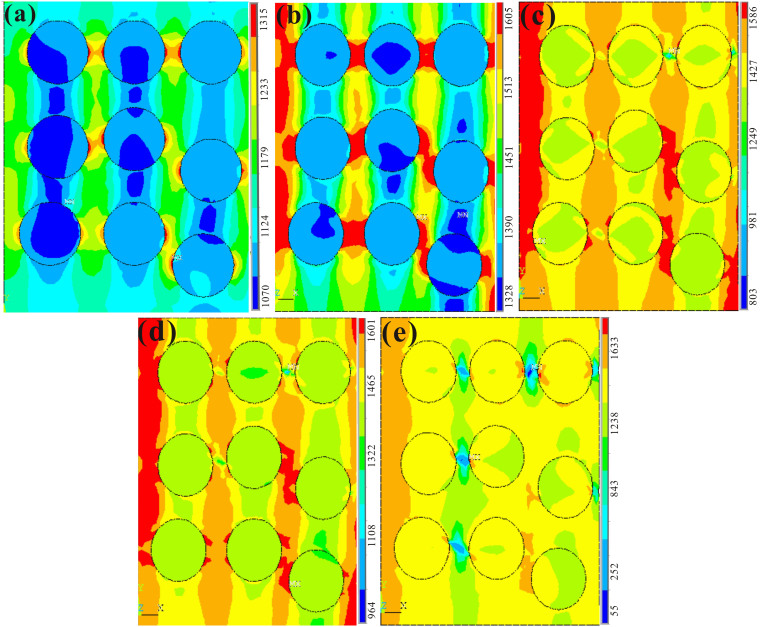
The contour maps of the stress distribution at the different strains in different stages: 1, 1.27, 1.35, 2, and 4% in (a), (b), (c), (d), and (e), respectively.

Figure 4 shows the contour maps of the stress distribution at different strains in different stages. Note that an approximation of spherical crystalline phase instead of dendrites is suggested in the FEM model^37^,^38^. Properties of the dendrites and amorphous matrix used in FEM are obtained from the calculations in this study and previous studies. Figure 4(a) shows the stress field within the composite at a strain of 1%. It clearly shows the stress concentrations at the interface. This results is due to the a disparity of the elastic limit, yield strength, and Young's modulus between the dendrites and amorphous matrix^1^,^6^,^19^, which consequently results in the stress concentration at the interface. The maximum stress within the composite is about 1288 MPa, which is lower than the yield stresses of both phases. Figure 4(b) depicts the stress distribution at a strain of 1.27%. It is indicated that the stress within the composite is obviously higher than that at a strain of 1%, and the maximum stress concentration among the dendrites can be found, consistent with previous reports^35^. The stress of the dendrites reaches the yielding stress, while the amorphous matrix is at the state of elastic deformation, in agreement with the deformation of the second stage in the current model.

As the deformation continues, the deformation of the composite enters into the plastic-plastic deformation stage. Figure 4(c) shows the shear-stress field at a strain of 1.35%, corresponding to the third stage. Obviously both phases deform plastically, since the stresses are beyond the yielding stress of both phases based on the estimation^21^,^22^. It has been demonstrated that deformation bands are formed inside dendrites in one parallel direction, and propagate into adjacent dendrites through the amorphous matrix, resulting in the availability of the maximum stress concentration occurring among the ductile dendrites^22^. Figures 4(d) and 4(e) present the stress field at strains of 2% and 4%, respectively. After large plastic deformation, the stresses within dendrites and near the interface are less than 100 MPa, as shown in Figure 4(e), revealing the alleviation of stress concentration during softening.

For comparison, the stresses in the dendrites, amorphous matrix, and composite obtained by the experiment, calculation, and FEM results are summarized in Table 1. The FEM simulation results are in good agreement with the experimental findings and theoretical calculations at each stage, indicating the consistence of the proposed model. The study reveals that the five-stage classification according to the mechanical behavior provides a crucial clue to elucidate the tensile deformation mechanisms.

*In-situ* dendrite/metallic glass matrix composites (MGMCs) with a composition of Ti_46_Zr_20_V_12_Cu_5_Be_17_, and a volume fraction of the dendrites of 57%, and a 2 μm average size of the composite, exhibit an ultimate tensile strength of 1510 MPa and a fracture strain of about 7.6%. The true stress-strain curves of the composite under tension are similar to those of the traditional carbon steels. The deformation behavior of the present composite can be classed into five stages: (1) elastic-elastic, (2) elastic-plastic, (3) plastic-plastic (yield platform), (4) plastic-plastic (work hardening), and (5) plastic-plastic (softening) stages. The constitutive relationships proposed at each deformation stage give the mathematical analysis on the deformation behavior, revealing the tensile deformation mechanism. The FEM simulations based on theoretical calculations confirm the theoretical model. Synthetically, experimental results, theoretical calculations, and FEM simulations are in good agreement with one another.

## Methods

The present Ti-based *in-situ* composites had a normal composition of Ti_46_Zr_20_V_12_Cu_5_Be_17_ (atomic percentage). The composite was prepared by arc-melting the mixture of high-purity element metals, Ti, Zr, V, Cu, and Be, in a Ti-getter high-purity argon atmosphere. The rods of 6 mm in diameter were produced using the copper-mold suction-casting method. The as-cast samples were characterized by high-energy synchrotron X-ray [111D-C, of the Advanced Photon Source (APS), Argonne National Laboratory (ANL), USA], scanning electron microscopy (SEM), and differential scanning calorimetry (DSC). DSC measurements were performed in a flowing argon atmosphere at a heating rate of 20 K/min. The Young's modulus of the amorphous matrix and dendrites was obtained by a Nano Indenter G200 with a strain rate of 0.05 s^−1^. The composites were machined into dog-bone-like rod specimens, which had a nominal gage diameter of 2 mm and gage length of 15 mm. The mechanical properties were characterized under quasi-static tension at a strain rate of 5 × 10^−4^ s^−1^. Finite element analysis was performed, using a commercial FEM software package, ANSYS 12.0. A free meshing method was adopted to mesh the model with mesh refinement near the interface of the dendrites and the matrix. Approximately 13000 elements were generated to represent the composites. Plane-strain calculations were applied. No slip boundary condition was used for the interface in the FEM analysis.

## Author Contributions

J.W.Q., T.Z. and B.S.X. designed the experiments. T.Z. carried out the experiments. J.W.Q., F.Q.Y., S.P. and P.K.L. analyzed the data, and J.W.Q. and T.Z. wrote the paper.

## Figures and Tables

**Table 1:** The stresses of the dendrites, amorphous matrix, and composite obtained by the experiment, calculation, and FEM results

	Experiment (MPa)			Calculation (MPa)			FEM (MPa)		
Strain	Dendrite	Matrix	Composite	Dendrite	Matrix	Composite	Dendrite	Matrix	Composite
1%	-	-	1144	1063	1304	1166	1081	1210	1131
1.27%	-	-	1450	1343	1656	1477	1353	1560	1448
1.35%	-	-	1460	1359	1603	1464	1374	1482	1459
2%	-	-	1498	1468	1538	1498	1406	1467	1491
4%	-	-	1430	1696	1079	1430	1411	1435	1420
